# Contributions of the paraventricular thalamic nucleus in the regulation of stress, motivation, and mood

**DOI:** 10.3389/fnbeh.2014.00073

**Published:** 2014-03-11

**Authors:** David T. Hsu, Gilbert J. Kirouac, Jon-Kar Zubieta, Seema Bhatnagar

**Affiliations:** ^1^Department of Psychiatry and the Molecular and Behavioral Neuroscience Institute, University of MichiganAnn Arbor, MI, USA; ^2^Departments of Oral Biology and Psychiatry, Faculties of Dentistry and Medicine, University of ManitobaWinnipeg, MB, Canada; ^3^Department of Anesthesiology, Children’s Hospital of Philadelphia, University of Pennsylvania Perelman School of MedicinePhiladelphia, PA, USA

**Keywords:** paraventricular, thalamus, subgenual, stress, anxiety, addiction, depression, orexin

## Abstract

The purpose of this review is to describe how the function and connections of the paraventricular thalamic nucleus (Pa) may play a role in the regulation of stress and negative emotional behavior. Located in the dorsal midline thalamus, the Pa is heavily innervated by serotonin, norepinephrine, dopamine (DA), corticotropin-releasing hormone, and orexins (ORX), and is the only thalamic nucleus connected to the group of structures comprising the amygdala, bed nucleus of the stria terminalis (BNST), nucleus accumbens (NAcc), and infralimbic/subgenual anterior cingulate cortex (sgACC). These neurotransmitter systems and structures are involved in regulating motivation and mood, and display abnormal functioning in several psychiatric disorders including anxiety, substance use, and major depressive disorders (MDD). Furthermore, rodent studies show that the Pa is consistently and potently activated following a variety of stressors and has a unique role in regulating responses to chronic stressors. These observations provide a compelling rationale for investigating the Pa in the link between stress and negative emotional behavior, and for including the Pa in the neural pathways of stress-related psychiatric disorders.

## Introduction

Stressful life events can facilitate and exacerbate anxiety disorders (ADs), substance use disorder (SUD), and major depressive disorder (MDD; Hammen, 2005; Andersen and Teicher, 2009; Nugent et al., 2011). Identifying a neuronal pathway by which stressors can influence motivation and mood is critical to understanding the development and maintenance of these disorders. In this review, we focus on the paraventricular nucleus of the thalamus (Pa) and its strong and specific connections with the amygdala, bed nucleus of the stria terminalis (BNST), nucleus accumbens (NAcc), and infralimbic/subgenual anterior cingulate cortex (sgACC) as shown in rodent and nonhuman primate studies. These connections represent pathways by which the Pa, strongly activated by a wide variety of stressors, may influence structures that regulate motivation and mood. Evidence from animal models suggest that peptidergic innervation of the Pa may play a role in anxiety and drug relapse, and the role of the Pa in chronic stress is discussed in relation to possible contributions to MDD. In addition, emerging neuroimaging studies suggest that it is possible to study the function of the Pa in humans.

## Anatomy and connections of the paraventricular nucleus of the thalamus (Pa)

### Anatomy

**Figure 1:**
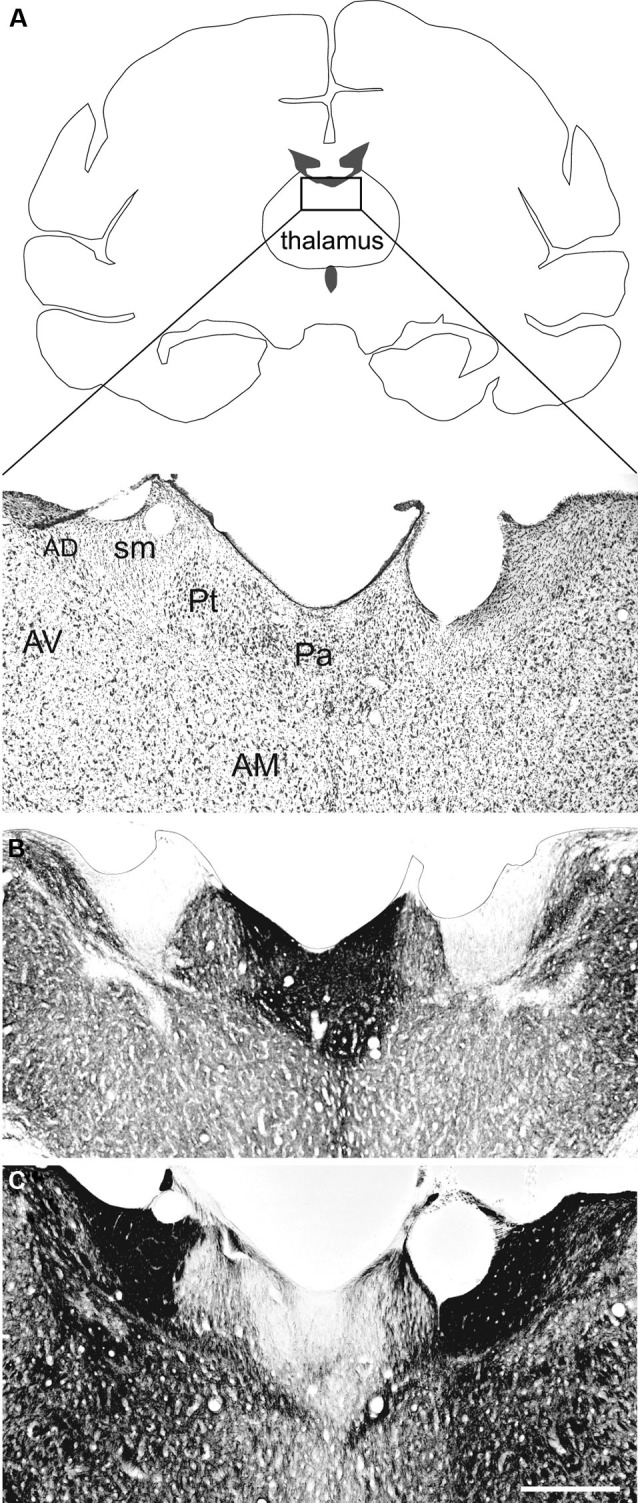
**The paraventricular nucleus of the thalamus (Pa) in nonhuman primates**. The Pa is a small group of densely-packed neurons in the dorsal midline thalamus. Shown here are three adjacent coronal sections through the dorsal midline thalamus of the *Macaca fascicularis* stained for ** (A)** Nissl, ** (B)** AChE, and ** (C)** myelin. The Pa is densely stained with AChE but is relatively lightly stained for myelin. Other thalamic nuclei shown are the anterodorsal (AD), anteromedial (AM), anteroventral (AV), and parataenial (Pt) nuclei. The stria medullaris (sm) is also shown. Scale bar = 500 µm. Adapted from Hsu and Price (2007)

The Pa is an elongated nucleus composed of small, densely packed neurons spanning the anterior-posterior extent of the dorsal midline thalamus (Figure 1A). It can be distinguished from surrounding nuclei by its moderate to dense staining for acetylcholinesterase and light staining for myelin both in monkeys (Figures 1B, C) and humans (Ohye, 1990; Uroz et al., 2004). An anterior and posterior portion has been described in animal models (Moga et al., 1995; Jones, 2007; Vertes and Hoover, 2008; Li and Kirouac, 2012), however this distinction is not easily identified in humans (Uroz et al., 2004; Jones, 2007). Some classify the Pa as part of the epithalamus, which includes the habenular nuclei and pineal body (Jones, 2007), while others include it in the midline and intralaminar group of thalamic nuclei (Morel et al., 1997).

An outline of Pa connections in rats and monkeys is described below. Across species, Pa connections are nearly identical except in specific structures in which the relative strength of connections differs. For example, in rats the Pa has strong projections to the central and basolateral amygdala (CeA, BLA; Moga et al., 1995; Li and Kirouac, 2008; Shin et al., 2008; Vertes and Hoover, 2008), whereas in monkeys the Pa projects primarily to the basal amygdala, with weak projections to the CeA (Hsu and Price, 2009). Differences in the connections between the anterior and posterior parts of the Pa (aPa, pPa) have also been shown in rats (Moga et al., 1995; Li and Kirouac, 2008; Vertes and Hoover, 2008), however it is not known if these differences exist in other species.

### Inputs to the paraventricular nucleus of the thalamus (Pa)

A recent study in rats using restricted iontophoretic tracer injections showed that the heaviest inputs to the Pa come from the prelimbic, infralimbic [areas comparable to cortical areas 32 and 25, respectively, in monkeys and humans (Öngür et al., 2003)] and agranular insular cortices, as well as the hippocampal subiculum (Li and Kirouac, 2012). This pattern of Pa input corresponds to that found in macaque monkeys (Hsu and Price, 2007, 2009). In both rats and monkeys the Pa receives significant input from the hypothalamus, particularly the dorsomedial nucleus, as well as from the lateral hypothalamic area, suprachiasmatic, and arcuate nucleus (Watts and Swanson, 1987; Chen and Su, 1990; Hsu and Price, 2009; Li and Kirouac, 2012). In both rats and monkeys the Pa also receives dense innervation from the periaqueductal gray (PAG; Krout and Loewy, 2000; Hsu and Price, 2009; Li and Kirouac, 2012), a pivotal site for coordinating visceral and behavioral responses related to pain and other stressors (Keay and Bandler, 2001), and the parabrachial nucleus (PB; Hsu and Price, 2009; Li and Kirouac, 2012). Other inputs to the Pa in both rats and monkeys include the entorhinal cortex, intergeniculate leaflet of the ventral lateral geniculate nucleus, zona incerta, amygdala, and BNST (Hsu and Price, 2009; Li et al., 2011; Li and Kirouac, 2012). In rats, it has also been shown that the aPa receives more inputs from the hippocampal subiculum and prelimbic cortex compared to the pPa, which receives relatively more inputs from the prelimbic, infralimbic, and agranular insular cortices (Li and Kirouac, 2012).

It is notable that the Pa is densely innervated by neurotransmitter systems that are involved in the response to stressors and implicated in ADs, SUD, and MDD. These inputs include the serotonin, dopamine (DA), norepinephrine, corticotropin-releasing hormone (CRH), orexins (ORX), and the endogenous opioids in rat, monkey, and human studies (Otake and Nakamura, 1995; Uroz et al., 2004; Kirouac et al., 2005; García-Cabezas et al., 2007; Jones, 2007; Vogt et al., 2008; Hsu and Price, 2009; Vertes et al., 2010). In particular, the Pa in rats and monkeys contains among the highest concentration of ORX fibers in the brain (Peyron et al., 1998; Kirouac et al., 2005; Hsu and Price, 2009). Thus, the Pa receives strong inputs from neurotransmitter systems and structures (e.g., PAG, BNST) that are activated in response to stressors, potentially transmitting these signals to its outputs.

### Outputs from the paraventricular nucleus of the thalamus (Pa)

Along with other midline and intralaminar thalamic nuclei, the Pa was long believed to project diffusely or nonspecifically to the cerebral cortex, in contrast to the cortical specificity exhibited by most of the other thalamic nuclei. However, experiments using modern tracing techniques have made it clear that the Pa has specific cortical projections. Studies in rats have shown that the Pa has strong projections to the infralimbic cortex (Berendse and Groenewegen, 1991; Moga et al., 1995; Li and Kirouac, 2008; Vertes and Hoover, 2008). Correspondingly, studies in macaque monkeys show that the Pa projects strongly to the cortex below the genu of the corpus callosum (i.e., sgACC; Hsu and Price, 2007, 2009), which has been implicated in sadness and depression in humans (Drevets et al., 1997; Hamani et al., 2011). Other cortical targets of the Pa in rats and monkeys include the dorsal agranular insular and entorhinal cortices (Berendse and Groenewegen, 1991; Moga et al., 1995; Hsu and Price, 2007; Li and Kirouac, 2008; Vertes and Hoover, 2008).

The Pa, which uses glutamate as a neurotransmitter (Frassoni et al., 1997), is unique from other midline and intralaminar nuclei in that it sends heavy projections to the shell of the NAcc in both rats and monkeys (Berendse and Groenewegen, 1990; Moga et al., 1995; Pinto et al., 2003; Li and Kirouac, 2008; Hsu and Price, 2009). In rats, these projections were shown to terminate onto dendritic spines in close proximity to dopaminergic terminals within the NAcc shell (Pinto et al., 2003; Parsons et al., 2007), and may regulate DA levels in the NAcc shell (Parsons et al., 2007). The Pa also projects strongly to the CeA, BLA, and BNST in rats (Moga et al., 1995; Li and Kirouac, 2008; Shin et al., 2008; Vertes and Hoover, 2008); and the basal nucleus of the amygdala and BNST in monkeys (Hsu and Price, 2009). It is interesting to note that the adjacent mediodorsal thalamic nucleus does not project to the NAcc, amygdala, or BNST in rats, cats, or monkeys (Russchen, 1982; Su and Bentivoglio, 1990; Giménez-Amaya et al., 1995; Li and Kirouac, 2008; Shin et al., 2008). In summary, the Pa has the distinction of being the only thalamic nucleus projecting to the group of structures comprising the amygdala, BNST, NAcc, and infralimbic/sgACC. These limbic structures are known for their roles in fear, anxiety, and reward behavior in animal models, and display abnormal activity in ADs, SUD, and MDD in humans (e.g., Drevets et al., 1997; Davis et al., 2010; Hamani et al., 2011; Blackford and Pine, 2012; Volkow et al., 2012; Berridge and Kringelbach, 2013; Jasinska et al., 2014). As described below, the Pa may play a role in transmitting and regulating stress-related information to these structures.

The aPa and pPa differ in their projection pattern, as shown in rats. The aPa has a widespread pattern of projection to the suprachiasmatic nucleus (SCN), dorsomedial and ventromedial hypothalamic nuclei, lateral septum, the BNST, CeA and basomedial amygdala, anterior olfactory nucleus, olfactory tubercle, NAcc, infralimbic, piriform, and perirhinal cortices, ventral subiculum, and endopiriform nucleus (Moga et al., 1995). In contrast, the pPa has a more restricted pattern of projection including the anterior olfactory nucleus, olfactory tubercle, NAcc, as well as stronger projections to the CeA, BLA, basomedial amygdala, lateral BNST, and interstitial nucleus of the posterior limb of the anterior commissure (Moga et al., 1995; Li and Kirouac, 2008; Vertes and Hoover, 2008). Potential differences between the projection pattern of the aPa and pPa have not been examined in other species.

**Figure 2:**
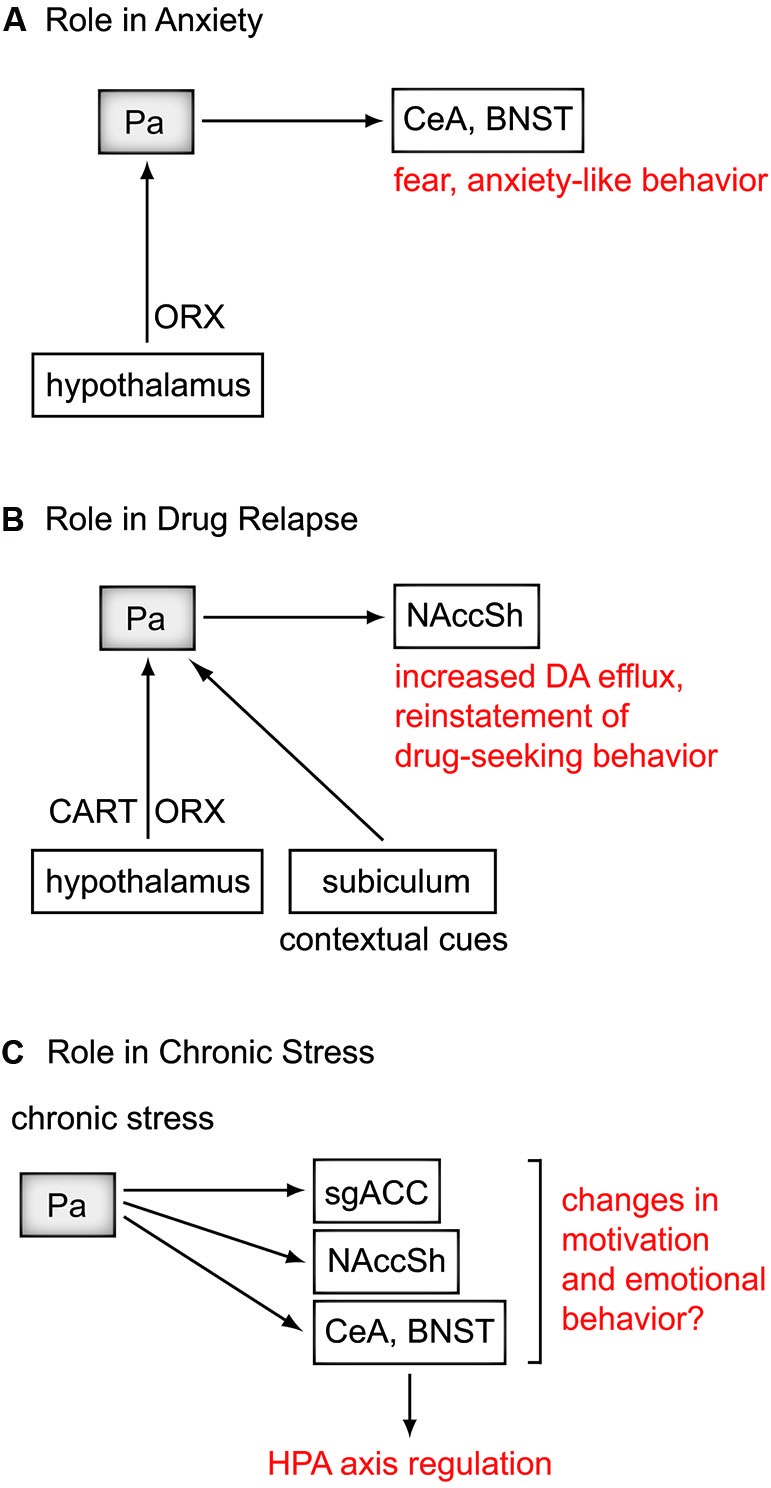
**Contributions of the Pa in psychiatric disorders**. Rodent studies suggest a role for the Pa in anxiety, drug relapse, and regulating the effects of chronic stress. **(A)** Orexin (ORX) projections from the hypothalamus to the Pa regulate fear and anxiety-like behavior through the central nucleus of the amygdala (CeA), and bed nucleus of the stria terminalis (BNST). **(B)** In a pathway for drug relapse, ORX and cocaine- and amphetamine-related transcript (CART) from the hypothalamus, and contextual cues from the subiculum project to the Pa. In turn, the Pa regulates dopamine (DA) efflux in the nucleus accumbens shell (NAccSh) and drug-seeking behavior. **(C)** Via the CeA and BNST, the Pa has been shown to be an important regulator of the hypothalamic-pituitary-adrenal (HPA) axis during chronic stress. A few studies in rodent models of depression have shown involvement of the Pa, however its specific role in depressive-like behavior remains to be determined. The role of the Pa in regulating chronic stress may also influence **(A)** and **(B)**. There is likely significant overlap between the pathways for these disorders, which are highly comorbid, and are exacerbated by severe or chronic stress. MDD, major depressive disorder; sgACC, subgenual anterior cingulate cortex.

**Figure 3:**
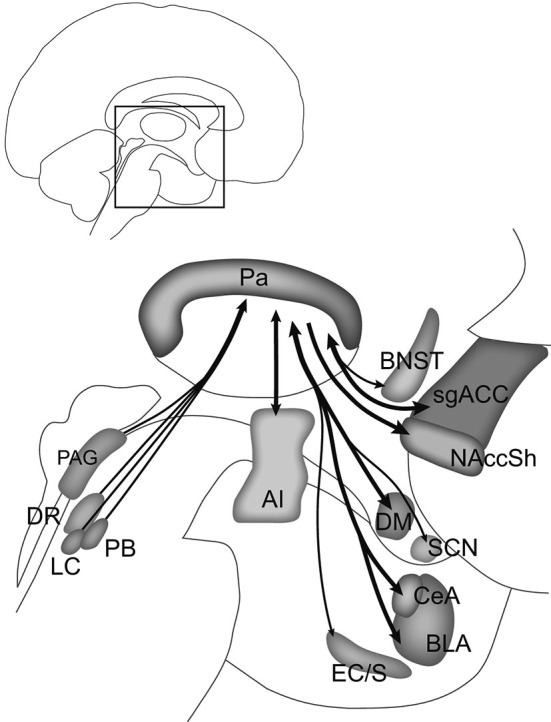
**Summary of Pa connections linking stress with motivation and mood**. The connections shown here in the human brain are based on rodent and nonhuman primate studies. Structures in the midbrain (periaqueductal gray, PAG; dorsal raphe, DR; locus coeruleus, LC; parabrachial nucleus, PB) send stress signals to the Pa, which in turn may influence activity in the agranular insular (AI) cortex, central (CeA) and basolateral (BLA) nuclei of the amygdala, nucleus accumbens shell (NAccSh), bed nucleus of the stria terminalis (BNST), and subgenual anterior cingulate cortex (sgACC), all of which are implicated in major depressive, substance use, and anxiety disorders. Other connections with the Pa include the entorhinal cortex/subiculum (EC/S), dorsomedial (DM) hypothalamus, and suprachiasmatic nucleus (SCN). Heavy input to the Pa by serotonin, norepinephrine, dopamine, corticotropin-releasing hormone, ORX, and endogenous opioids may regulate this pathway and determine how stressors, particularly chronic stressors, influence motivation and mood. Inset shows area of detail in the human brain. Adapted from Hsu and Price (2009).

In both rats and monkeys, the Pa is reciprocally connected with the SCN (Watts and Swanson, 1987; Hsu and Price, 2009), the brain’s circadian pacemaker, and has been investigated for its role in the entrainment of circadian rhythms to light in rats (Salazar-Juárez et al., 2002). For example, Pa neurons that follow a circadian rhythm project to the amygdala and NAcc (Peng et al., 1995), and the Pa may provide the relay by which the SCN influences amygdala and prefrontal cortical activity (Sylvester et al., 2002; Peng and Bentivoglio, 2004). The Pa has also been shown to be necessary in reducing the amplitude of circadian body temperature rhythms during chronic stress (Bhatnagar and Dallman, 1999). Disturbed circadian rhythms are strongly associated with MDD, and normalize following successful treatment (McClung, 2007). Thus the Pa, with its input from the SCN and strong connections with mood-regulating structures, may provide a link between disturbances in circadian rhythms and disturbances in mood. Pa pathways involved in animal models of psychiatric disorders are shown in Figure 2, and a summary of the major connections of the Pa mapped onto the human brain is shown in Figure 3.

## Role of the paraventricular nucleus of the thalamus (Pa) in acute and chronic stress

### Sensitivity to acute stress and emotional arousal

In rats, the Pa is consistently and strongly activated following a wide variety of stressors including conditioned fear, restraint, handling, swim, mild footshock, air puff, and sleep deprivation (Sharp et al., 1991; Beck and Fibiger, 1995b; Cullinan et al., 1995; Bhatnagar and Dallman, 1998; Bubser and Deutch, 1999; Semba et al., 2001; Otake et al., 2002; Spencer et al., 2004). This suggests that the Pa is part of a common pathway that is activated regardless of the stressor type. For example, a dual immunohistochemistry study showed that acute immobilization stress induces Fos protein expression in Pa-projecting neurons in several areas known to be involved in the response to stressors, including the PAG, locus coeruleus (LC), dorsal raphe (DR), PB, nucleus of the solitary tract, and ventrolateral medulla (Otake et al., 2002). In turn, stress-activated Pa neurons project to the CeA/BLA of the amygdala, NAcc, and the medial prefrontal cortex following mild footshock (Bubser and Deutch, 1999), or to the CeA following forced swim (Zhu et al., 2011). The Pa may influence activity in these structures by increasing DA utilization in the NAcc (Jones et al., 1989; Parsons et al., 2007), and dampening activity in the CeA during acute stress (Spencer et al., 2004), possibly by activating inhibitory local circuits within the CeA (Veinante and Freund-Mercier, 1998).

Accumulating evidence also indicate that the Pa is activated in the context of emotionally arousing environments, both aversive and rewarding. For example, Pa neurons are activated after rats are exposed to cues signalling a sweetened water reward (Igelstrom et al., 2010), or when placed in a context associated with a taste aversion (Yasoshima et al., 2007), drug reward (Hamlin et al., 2009; James et al., 2011), or footshock (Beck and Fibiger, 1995b; Yasoshima et al., 2007). This suggests that information associated with emotionally charged events is transmitted to the Pa, which then provides excitatory inputs to the amygdala, NAcc, prefrontal cortex, and other areas of the forebrain involved in the expression of both positive and negative emotional states (Hamlin et al., 2009; Zhu et al., 2011). Consistent with this view, lesions of the Pa or inactivation of neurons in the Pa have been shown to attenuate the expression of emotionally charged behavioral responses such as conditioned taste aversion (Yamamoto et al., 1995), locomotor response to a cocaine-paired environment (Young and Deutch, 1998), cocaine-induced conditioned place preference (Browning et al., 2014), drug-seeking behavior following cocaine-priming (James et al., 2010), and context-induced reinstatement of extinguished alcohol-seeking behavior (Hamlin et al., 2009). While data are accumulating in support of a role for the Pa in both negatively and positively charged emotional behavior, the mechanisms by which the Pa can be involved in behavioral responses with opposite emotional directions remains to be determined.

## Peptidergic innervation of the paraventricular nucleus of the thalamus (Pa)

### Role in anxiety

There is growing interest in the potential importance of the intense hypothalamic peptidergic inputs to the Pa (Freedman and Cassell, 1994; Kirouac et al., 2005, 2006). In particular, the Pa receives among the densest ORX input in the brain in rodents and nonhuman primates (Peyron et al., 1998; Kirouac et al., 2005; Hsu and Price, 2009). ORX has functions related to stress (Berridge et al., 2010) and is likely to exert some of these functions by depolarizing Pa neurons (Ishibashi et al., 2005). Recent studies show that stimulation of ORX receptors in the region of the Pa produces fear and anxiety-like behaviors in rats (Li et al., 2009, 2010a,b; Heydendael et al., 2011), and blockade of ORX receptors in the Pa has anxiolytic effects (Li et al., 2010b) and prevents facilitation of the hypothalamic-pituitary-adrenal (HPA) axis to novel stress following repeated stress (Heydendael et al., 2011). Other studies show that blocking ORX receptors in the Pa attenuates the expression of negative emotional states associated with morphine withdrawal (Li et al., 2011). ORX in the Pa may exert these effects through CeA projections, which has been shown to be involved in the retrieval of well consolidated fear memories (Padilla-Coreano et al., 2012), or through projections to the BNST (Li and Kirouac, 2008), a region implicated in ADs. Thus, ORX projections to the Pa may play a role in regulating the expression of anxiety (Figure 2A).

### Role in drug relapse

Preventing relapse is perhaps the most difficult aspect of treating drug addiction (O’Brien, 2005), and accumulating evidence suggests that the Pa is involved in sensitization to drug-associated environmental cues. The Pa is activated to contextual cues to a methamphetamine or cocaine-paired environment (Rhodes et al., 2005; James et al., 2011), and lesions of the Pa block the conditioned locomotor response to a cocaine-paired environment (Young and Deutch, 1998). The Pa-NAcc pathway has also been shown to be involved in the context-induced reinstatement of seeking alcoholic beer (Hamlin et al., 2009). The Pa may receive context-related information processed by the subiculum (Chen and Su, 1990; Hsu and Price, 2009; Li and Kirouac, 2012) and mediate reinstatement behavior by regulating DA release in the NAcc (Jones et al., 1989; Pinto et al., 2003; Parsons et al., 2007), or through other structures involved in relapse such as the BLA and medial prefrontal cortex (Schmidt et al., 2005).

A recent review suggests that ORX transmission in the Pa may play a role in relapse (Martin-Fardon and Boutrel, 2012). In addition, the cocaine- and amphetamine-related transcript (CART), another peptide that densely innervates the Pa (Kirouac et al., 2006), may act together with ORX to regulate the rewarding effects of drugs of abuse. Microinjections of the CART peptide into the Pa region attenuated cocaine seeking in drug-primed rats (James et al., 2010). In addition, increased Fos-positive neurons were found in the Pa during environmental cues for ethanol, and were closely associated with ORX and CART terminal fields (Dayas et al., 2008). Thus, ORX and CART input to the Pa may play a central role in drug relapse by regulating responses to drug-related cues (Figure 2B).

### Role in chronic stress and vulnerability to major depressive disorder (MDD)

Perhaps the most intriguing aspect of the Pa is that it plays a unique role in regulating neuroendocrine and behavioral adaptations to severe or chronic stress (Bhatnagar and Dallman, 1998; Bhatnagar et al., 2000, 2002, 2003). Rats that have experienced several days of repeated stress show habituation of the HPA axis to a subsequent stressor if it is of the same type as the repeated stressor (Bhatnagar et al., 2002). However, response of the HPA axis is often facilitated or enhanced if the subsequent stressor is of a different type from the repeated stressor, possibly reflecting the ability to overcome negative feedback effects of glucocorticoids released by prior chronic stress, and respond to a novel stressor that poses a potential threat to survival (Bhatnagar and Dallman, 1998). During chronic stress, an intact pPa is necessary for both habituation and facilitation of the HPA axis (Bhatnagar and Dallman, 1998; Bhatnagar et al., 2000, 2002). Interestingly, the pPa is not engaged in the regulation of HPA responses to an acute stressor that was not preceded by repeated stress (Bhatnagar and Dallman, 1998; Bhatnagar et al., 2000, 2002). Although these studies were conducted in adult animals, the Pa is activated by recurrent handling but not by acute handling in P9 rat pups (Fenoglio et al., 2006), suggesting that the specific engagement of the Pa in conditions of chronic stress or stimulation may occur throughout the lifespan.

Recent studies have yielded a more nuanced picture revealing that the pPa is critical for the development of habituation and facilitation, however, once developed the expression of habituation and facilitation do not seem to be regulated by the pPa. The role of the pPa in chronic stress may be regulated by glucocorticoid actions in the Pa (Jaferi et al., 2003; Jaferi and Bhatnagar, 2006), ORX (Heydendael et al., 2011), ORX influencing the gene expression of the CRH receptor 1 (CRHR1) receptor (Heydendael et al., 2012), or alpha-2B adrenoceptors, which have been shown to be upregulated specifically in the Pa following chronic psychosocial stress in tree shrews (Heilbronner et al., 2004). The pPa has few if any direct projections to the paraventricular nucleus (PVN) of the hypothalamus (Moga et al., 1995; Li and Kirouac, 2008), a structure critical for initiating the HPA axis response (Sawchenko et al., 1996), and may instead regulate the HPA axis via multisynaptic pathways through the BNST to the PVN (Prewitt and Herman, 1998).

The role of the Pa in chronic stress sheds light on the poorly understood relationship between stress and the onset of MDD. It has been hypothesized that chronic stressors exacerbate the effects of acute stressors, or conversely an acute stressor can magnify the depressive consequences of chronic stressors (Hammen, 2005). The involvement of the Pa may depend on whether the acute stressor leads to habituation or facilitation. For example, acute stressors can facilitate the occurrence of MDD in the presence of high levels of chronic stress (Hammen et al., 2009). It is possible that a dysregulated HPA axis that is often associated with MDD is in part an inability for the Pa to respond adaptively to chronic stress. Furthermore, an abnormal Pa in MDD may exacerbate or sustain the negative emotional effects of chronic stress by contributing to elevated sgACC activity. This possibility is supported by the observation that the “medial” thalamus including the Pa exhibits greater functional connectivity with the sgACC in patients with MDD compared to controls (Greicius et al., 2007). Abnormal Pa activity may also influence the NAcc, and may be responsible for anhedonia in MDD, and/or drug reinstatement in SUD (Pizzagalli et al., 2009; Millan et al., 2011). Thus, the Pa may play a role in mediating the relationship between chronic stress and MDD (Figure 2C).

The Pa is a key structure in multiple rodent models of MDD. Following forced swimming, increased levels of c-fos in the Pa was correlated with levels of immobility, and Pa neurons projecting to the CeA showed increased levels of c-fos, suggesting a role for the Pa-CeA pathway in depressive-like behavior (Zhu et al., 2011). Following repeated exposure to uncontrollable footshock stress, the Pa was one of only five structures (out of 60 brain areas surveyed) with reduced c-fos levels following chronic administration of desipramine (a tricyclic antidepressant), which also resulted in reduced depressive-like behaviors (Beck and Fibiger, 1995a). In olfactory bulbectomized rats, a validated model of MDD (Song and Leonard, 2005), chronic fluoxetine treatment reduced depressive-like behavior in the open-field test, and reduced c-fos expression in the Pa, amygdala, hippocampus, and DR nucleus (Roche et al., 2007). In a rodent study of repetitive transcranial magnetic stimulation (rTMS) as a potential treatment for refractory MDD, the greatest increase in c-fos levels was in the Pa (Ji et al., 1998). Taken together, these studies suggest that the Pa is sensitive to multiple rodent models of MDD and MDD treatments. Future studies specifically targeting the Pa with lesions or pharmacological manipulations are needed to examine the specific role of the Pa in rodent models of MDD.

### Human studies of the paraventricular nucleus of the thalamus (Pa)

The small size of the Pa makes it difficult to identify in neuroimaging studies, however a few studies strongly suggest receptor binding specifically in the Pa with positron emission tomography (PET) and activation of the Pa with functional magnetic resonance imaging (fMRI). For example, as described below, high levels of receptor binding specifically in the Pa in postmortem human brains (with high anatomical resolution) suggest that radioligand binding for that particular receptor in the medial thalamus detected with PET (with lower anatomical resolution) is likely in the Pa. We describe below the only human neuroimaging studies to our knowledge that have identified the Pa. Given the strong connections of the Pa to structures often showing abnormal neural activity in AD, SUD, and MDD (including the amygdala, NAcc, BNST, and sgACC), and its role in regulating stress, the Pa may be considered a key component of the neural pathways involved in these disorders (Figure 3).

### Positron emission tomography with radioligands

In humans, the Pa contains the highest density of [^125^I]epidepride D_2/3_-binding sites (2.5× more than that of the adjacent mediodorsal nucleus) in the thalamus as shown by *in situ* autoradiography (Rieck et al., 2004). This corresponds to PET data showing that the Pa has the highest thalamic binding potential *in vivo* for the D_2_ radioligand [^18^F]fallypride, whereas binding in the mediodorsal nucleus was significantly less (Rieck et al., 2004). Thus, DA binding in the Pa can be identified and studied in humans. The function of DA release into the Pa is not known, although D_2/3_ agonists have been shown to activate Pa neurons in rats (Deutch et al., 1998).

In human postmortem brains, high levels of 5-hydroxytryptamine 1a (5-HT1a) receptor binding were found in the midline thalamic nuclei, with low to absent binding in the mediodorsal thalamus (Varnäs et al., 2004). This finding is consistent with the pattern of binding found in the thalamus for 5-HT1 in macaque monkeys (Stuart et al., 1986). Interestingly, radiolabeled selective serotonin reuptake inhibitors (SSRIs) have been shown to accumulate to a high degree in the midline and dorsal thalamic nuclei including the Pa in pigs, monkeys, and humans (Smith, 1999), suggesting that serotoninergic neurotransmission to the Pa may be involved in the antidepressant effects of SSRIs.

It has been consistently shown in humans that the thalamus has the highest levels of µ-opioid receptor binding (Sprenger et al., 2005). The greatest acute reduction in µ-opioid receptor availability (reflecting increases in µ-opioid-mediated neurotransmission) following pain challenges was found in the medial thalamus, which includes the Pa (Zubieta et al., 2001; Bencherif et al., 2002). Furthermore, a recent study showed that social rejection (i.e., when one is not liked by others) also increased µ-opioid receptor-mediated neurotransmission in the midline thalamus that includes the Pa (Hsu et al., 2013). This study suggests that the µ-opioid receptor system, which alleviates physical pain, may also regulate “social pain” through the Pa. Consistent with the human data, the Pa in rats contains among the highest levels of µ-opioid receptor immunoreactivity in the thalamus (Ding et al., 1996) and contains the densest thalamic concentration of fibers for the endogenous µ-opioid receptor ligands endomorphin-1 and -2, enkephalin, and beta-endorphin, with few fibers in the surrounding mediodorsal nucleus (Sar et al., 1978; Coveñas et al., 1996; Martin-Schild et al., 1999; Uroz et al., 2004). Following µ-opioid receptor stimulation in rats, the Pa and other midline nuclei showed the highest activation in the thalamus as measured by c-Fos (Jiang et al., 2000) or [^35^S]GγS binding for detecting G-protein activation (Sim-Selley et al., 1999). Activation of µ-opioid receptors in the Pa may function to inhibit firing of neurons in the Pa (Brunton and Charpak, 1998), potentially alleviating the effects of stressors. It remains to be determined if activation of the µ-opioid receptor system in the Pa during physical pain and social rejection in humans regulates motivation and mood through Pa projections to the NAcc, amygdala, PAG, agranular insular (AI) cortex, and sgACC found in rodents and nonhuman primates (Berendse and Groenewegen, 1990, 1991; Moga et al., 1995; Pinto et al., 2003; Hsu and Price, 2007, 2009; Li and Kirouac, 2008; Vertes and Hoover, 2008).

### Functional magnetic resonance imaging

Although the spatial resolution used in most fMRI studies does not allow for the identification of the Pa beyond what can be called the “medial” thalamus, a few studies are worth mentioning. Following eight days of repeated experimental pain, peak levels of *decreased* activity were found in the medial thalamus including the Pa (peak: *x* = 0, *y* = −9, *z* = 6, Montreal Neurological Institute (MNI) coordinates) in healthy individuals (Bingel et al., 2007). This finding is consistent with the role of the Pa in regulating the effects of chronic stress in animal models as described above. Furthermore, in those who showed behavioral habituation to the repeated pain, the sgACC showed increased activity, suggesting that decreased Pa activity allowed for the sgACC to engage in mediating the habituation to pain (Bingel et al., 2007). The functional relationship between the Pa and sgACC was also highlighted in an fMRI study that examined functional connectivity (i.e., activity correlation) during rest. In this study, patients diagnosed with MDD showed greater resting state functional connectivity between the medial thalamus including the Pa, and the sgACC, compared to healthy controls (Greicius et al., 2007). These two studies suggest that chronic stressors may be regulated by the Pa-sgACC pathway, which may be overactive in MDD, potentially reflecting excessive coupling between the Pa and the “affective” sgACC, at the expense of reduced connectivity to the “cognitive” dorsal ACC (Anand et al., 2005a,b; Greicius et al., 2007). Future studies using higher resolution fMRI are needed to positively identify the Pa in its role in regulating chronic stress and related disorders. One study using high resolution fMRI (7 Tesla) identified activation in the Pa while men viewed sexual images (Metzger et al., 2010), showing that this level of analysis is possible.

## Conclusion

The Pa is positioned to influence limbic structures controlling motivation and mood. The Pa is sensitive to environmental stressors and cues for drugs of abuse and may mediate stress-induced changes in mood and behavior through the amygdala, BNST, NAcc, and sgACC. The Pa also plays an important role in the regulation of chronic stress through ORX inputs, although several neurotransmitter systems are likely involved. These observations bring the Pa into focus as a critical component in a pathway by which stressors, particularly chronic stressors, can influence motivation and mood, potentially promoting stress-related psychiatric disorders. Further investigation of the Pa in this role in animal models and in humans is warranted.

## Conflict of interest statement

The authors declare that the research was conducted in the absence of any commercial or financial relationships that could be construed as a potential conflict of interest.
